# Early impacts of climate change on a coastal marine microbial mat ecosystem

**DOI:** 10.1126/sciadv.abm7826

**Published:** 2022-05-27

**Authors:** Usha F. Lingappa, Nathaniel T. Stein, Kyle S. Metcalfe, Theodore M. Present, Victoria J. Orphan, John P. Grotzinger, Andrew H. Knoll, Elizabeth J. Trower, Maya L. Gomes, Woodward W. Fischer

**Affiliations:** 1 Division of Geological and Planetary Sciences, California Institute of Technology, Pasadena, CA 91125, USA.; 2 Department of Organismic and Evolutionary Biology, Harvard University, Cambridge, MA 02138, USA.; 3 Department of Geological Sciences, University of Colorado Boulder, Boulder, CO 80309, USA.; 4 Department of Earth and Planetary Sciences, Johns Hopkins University, Baltimore, MD 21218, USA.

## Abstract

Among the earliest consequences of climate change are extreme weather and rising sea levels—two challenges to which coastal environments are particularly vulnerable. Often found in coastal settings are microbial mats—complex, stratified microbial ecosystems that drive massive nutrient fluxes through biogeochemical cycles and have been important constituents of Earth’s biosphere for eons. Little Ambergris Cay, in the Turks and Caicos Islands, supports extensive mats that vary sharply with relative water level. We characterized the microbial communities across this variation to understand better the emerging threat of sea level rise. In September 2017, the eyewall of category 5 Hurricane Irma transited the island. We monitored the impact and recovery from this devastating storm event. New mat growth proceeded rapidly, with patterns suggesting that storm perturbation may facilitate the adaptation of these ecosystems to changing sea level. Sulfur cycling, however, displayed hysteresis, stalling for >10 months after the hurricane and likely altering carbon storage potential.

## Teaser

Microbial mats resembling Earth’s earliest ecosystems exhibited remarkable resilience following devastation by Hurricane Irma.

## INTRODUCTION

Coastal environments are uniquely vulnerable to the consequences of climate change; lying at the interface of land, ocean, and atmosphere, they are directly affected by sea level rise, extreme weather, and changes in both air and ocean temperature and chemistry. Some coastal ecosystems, e.g., coral reefs and salt marshes, are already suffering devastating losses in extent and biodiversity (*1*–*3*), while others, e.g., mangrove forests, appear to be expanding and even mitigating climate change impacts by enhancing land stabilization and carbon storage (*4*–*7*). Here, we examined a predominantly microbial ecosystem facing these challenges: photosynthetic microbial mats, which are often found in close association with mangroves and are thought to play a major role in shallow sediment nutrient availability (*8*).

Photosynthetic microbial mats are assemblies of microbes that form layered, macroscopic structures. Their fabric is commonly built by filamentous Cyanobacteria (*9*), and the communities that inhabit them rank among the most diverse microbial ecosystems known (*10*–*12*). Within a mat, steep physicochemical gradients partition a complex network of niche spaces (*13*, *14*)—sunlight drives phototrophy in the surface layers (*15*–*17*); in the subsurface, redox stratification and other chemical gradients support a wide range of anaerobic metabolisms (*18*–*23*)—and tightly coupled metabolic interactions fuel rapid and dynamic biogeochemical cycling with a diurnal cadence (*13*, *24*). These ecosystems have been important components of the biosphere since long before the rise of plants and animals (*25*–*27*), a history recorded by their mineralized vestiges preserved in ancient sedimentary rocks (*28*–*30*).

**Fig. 1.**
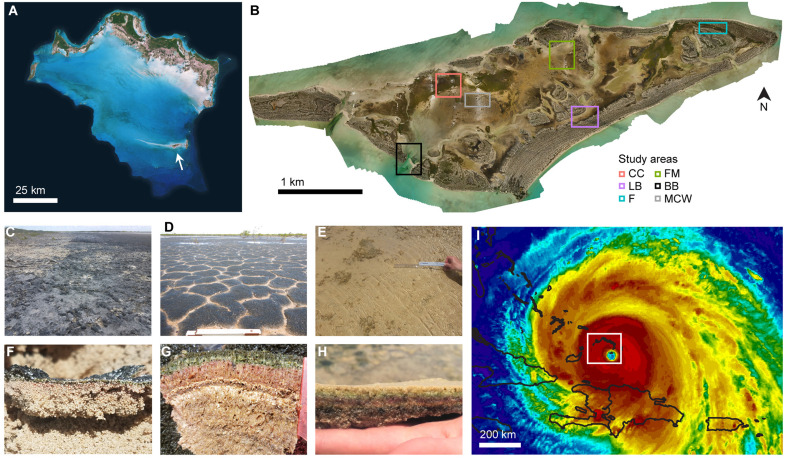
Maps and context images. (**A**) Satellite image of the Caicos carbonate platform, white arrow pointing out Little Ambergris Cay. (**B**) Drone orthomosaic of Little Ambergris Cay with study areas indicated. Aerial images of these regions documenting changes over time and sample details can be found in fig. S1. (**C** to **H**) Surface (C to E) and cross-sectional (F to H) photographs of end-member mat types—blister mats, of millimeter-scale thickness characterized by rough, black, or gray surfaces (C and F); polygonal mats, of centimeter- to decimeter-scale thickness with highly cohesive, often fibrous mat fabric and dark green tufted surfaces characterized by desiccation cracks that delineate polygons (D and G); and smooth mats, of generally centimeter-scale thickness and ranging in consistency from moderately cohesive to loose and goopy, often covered in beige exopolysaccharide material (E and H). (**I**) National Oceanic and Atmospheric Administration Geostationary Operational Environmental Satellite network infrared image of Hurricane Irma with the eye directly over Little Ambergris Cay on 7 September 2017, 22:45 UTC. Black traces indicate land masses, and white box indicates the area shown in (A).

Little Ambergris Cay is an uninhabited island in the Turks and Caicos with a broad, shallow interior basin widely paved by benthic microbial mats (Fig. 1, A and B). This remote environment is an ideal natural laboratory—both for better understanding modern mat ecosystems and as an analog for the ancient mat ecosystems that dominated the Earth through much of its history (*31*–*35*). The mats on Little Ambergris Cay exhibit a variety of macroscopic textures (Fig. 1), ranging in thickness from millimeters to decimeters, in consistency from leathery to gelatinous, and in surface character from botryoidal or tufted to smooth. In previous studies, these mats have been categorized into three end-member types (*32*), termed blister mats, polygonal [or biscuit (*31*)] mats, and smooth [or flat (*31*, *33*)] mats (Fig. 1, C to H). The basis for this morphological diversity has been of interest to the geobiological community, as mat textures preserved in the geological record provide clues about ancient microbial ecosystems (*31*, *32*).

Previously, we conducted a comprehensive mapping effort of these different mat types across Little Ambergris Cay and showed that the primary factors determining their distribution are water depth and tidal exposure time above water (*32*). The mats exist only within a narrow elevation range; areas higher than 30 cm above mean water level host scrubland rather than mat, and areas lower than 20 cm below mean water level strong hydrodynamic forces inhibit mat development. Within this range, blister mats occur in the highest, driest areas (subaerial exposure times of 22 to 24 hours/day), polygonal mats in intermediate areas (subaerial exposure times of 12 to 23 hours/day), and smooth mats in lower, wetter areas (subaerial exposure times of 0 to 12 hours/day). Since small (centimeter-scale) differences in water level exert such a strong control on mat habitat ranges, this is a system that is acutely sensitive to one of the most immediate consequences of global climate change—sea level rise (*36*). However, observations of ancient mat ecosystems from the geological record demonstrate that mats have persisted across numerous intervals of rising and falling sea level, with textural changes tracking changes in water depth (*37*, *38*). This history suggests the hypothesis that while mat ecosystems are finely tuned to water level, they may also be robustly adaptable.

The present study of Little Ambergris Cay microbial mat communities was initiated to better understand the ecological differences among mat types. Our approach combined 16*S* ribosomal RNA (rRNA) gene amplicon sequencing and community analysis with physical, geochemical, and biological field observations. Initial field campaigns were conducted in July 2016 and August 2017, surveying the diversity of microbial mats across the island in 2016 and focusing on the ecosystem structure with depth in 2017.

In September 2017, Little Ambergris Cay experienced a direct hit by the eyewall of category 5 Hurricane Irma (Fig. 1I)—one of the strongest hurricanes ever recorded in the Atlantic—with 920-mbar average atmospheric pressure and sustained 170 miles/hour winds accompanied by an estimated 3.2-m storm surge (*39*, *40*). Tropical cyclones of increasing intensity are another impending consequence of climate change (*41*). While sea level rise, warming, and acidification manifest over time scales of decades, extreme weather events cause marked environmental changes over time scales of hours to minutes and therefore can be much more immediately devastating to vulnerable ecosystems (*1*, *42*). In contrast to the adaptability of mat ecosystems to changes in sea level, the geological record demonstrates that sudden blanketing with a sediment layer can terminate mat growth (*28*). Having characterized the baseline ecosystem just before Hurricane Irma, we were uniquely well poised to investigate how the mat communities responded to such a catastrophic disturbance. Follow-up studies were conducted in March 2018, July 2018, and June 2019, to document the impact and subsequent recovery.

## RESULTS

### Profile of a microbial mat ecosystem

**Fig. 2.**
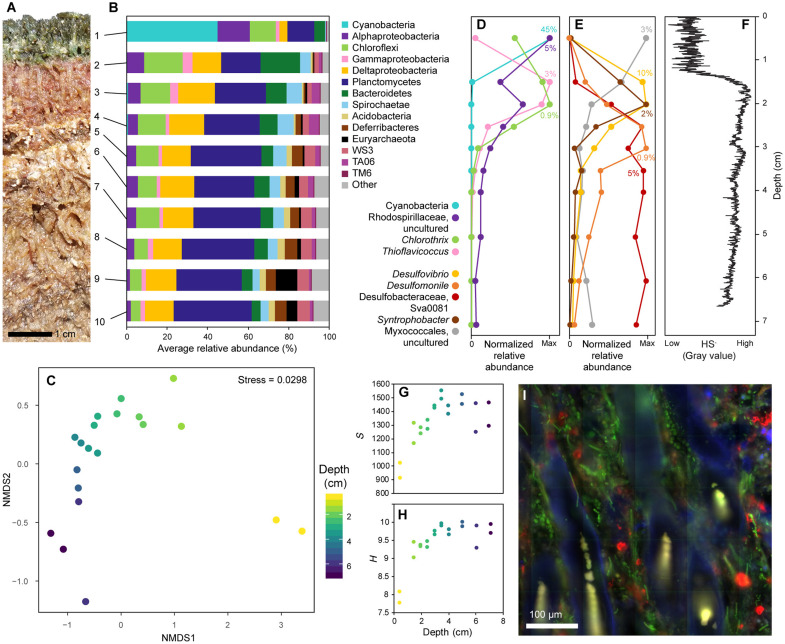
Microbial mat stratigraphy. (**A**) Photograph of CC mat cross section. Each visually distinguishable layer was sampled in replicate for microbial community analysis; the numbers to the right of the photograph indicate the horizons sampled. (**B**) Phylum level community composition of each layer; data shown are the averages of replicates. (**C**) NMDS plot showing variance in the microbial communities with depth. Each point represents a sample; relative proximity between points indicates similarity. Replicate samples are plotted separately, illustrating the minor amount of heterogeneity between replicates. (**D** and **E**) Normalized relative abundance showing the distribution of major groups of phototrophs (D) and Deltaproteobacteria (E) with depth, demonstrating the presence of an oxic photic zone, a sulfidic photic zone, and at least four distinct zones of organic carbon breakdown. Percentages indicate the abundance of each taxon relative to the full community, indicated at the horizon where their relative abundance peaks. (**F**) Sulfide profile captured on a silver strip, illustrating the porefluid chemocline from oxic to sulfidic ~1.3 cm below the mat surface. (**G**) Alpha diversity [observed operational taxonomic units (OTUs)] and (**H**) Shannon diversity [$H=-\Sigma_{i=1}^{s}(p_{i}{\text{log}}_{2}p_{i})$] of each sample with depth. (**I**) Fluorescence microscopy showing the complexity of spatial relationships and microenvironments in a mat slice. Red is fluorescence in situ hybridization (FISH) labeling of 16*S* rRNA with a universal bacterial probe; blue is 4′,6-diamidino-2-phenylindole (DAPI), a general DNA stain; and green is cyanobacterial autofluorescence.

To characterize the Little Ambergris mat ecosystems, we sampled a depth profile through a polygonal mat in the area designated CC, chosen for being among the lushest (~10-cm-thick, well-protected by mangroves) mat sites on the island (Fig. 2). The mat communities contained a diverse assemblage of organisms with abundant Cyanobacteria, Chloroflexi, Alphaproteobacteria, Gammaproteobacteria, Deltaproteobacteria, Planctomycetes, and Bacteroidetes, along with 40 additional phyla (Fig. 2B). Nonmetric multidimensional scaling (NMDS) ordination analysis to visualize dissimilarity showed that the surface community is notably distinct, while the subsurface layers displayed a gradual trend of variance with depth (Fig. 2C). The strong shift between surface (top centimeter) and subsurface (>1.5 cm) communities coincides with the transition from oxic to sulfidic conditions (Fig. 2F).

To understand community stratigraphy on a functional level, we examined select groups of organisms whose metabolisms can reasonably be inferred from taxonomy (Fig. 2, D and E). Among the phototrophs (Fig. 2D), sequences associated with oxygenic photosynthetic Cyanobacteria (including abundant members of the genera *Halomicronema*, *Calothrix*, and other unassigned Cyanobacteria) were recovered only in the uppermost horizon. Genera that likely represent facultatively aerobic anoxygenic photoheterotrophs (*Chlorothrix* of the Chloroflexales and an uncultured member of the Rhodospirillales) were also present at the surface but extended deeper in the mat than the Cyanobacteria. Sulfide-oxidizing anoxygenic phototrophs (*Thioflavicoccus* of the Chromatiales) were absent from the surface layer but found in a near subsurface horizon, co-occurring with the sulfidic chemocline. This distribution of phototrophs demonstrated the presence of both oxic and sulfidic regions within the photic zone of the mat and reflects the ability of different groups to use both different wavelengths of light that penetrate the mat to different extents and different electron donors that vary with depth (*15*), consistent with observations from previously characterized mat ecosystems (*13*).

Distinct heterotrophic guilds were illustrated by major deltaproteobacterial taxa, which included three genera of sulfate-reducing bacteria (*Desulfovibrio*, *Desulfomonile*, and Desulfobacteraceae group Sva0081), the genus *Syntrophobacter* (likely fermentative or syntrophic), and an uncultured member of the Myxococcales (aerobic) (*43*). The Myxococcales were most abundant in the oxic surface layer, while all four anaerobic genera were absent at the surface (Fig. 2E). This is notably different from other mat ecosystems wherein sulfate-reducing bacteria are closely associated with oxygenic Cyanobacteria in the surface layer (*44*). *Desulfovibrio* and *Syntrophobacter* appeared in the near subsurface, co-occurring with the sulfidic chemocline. About 3 cm below the surface, they were replaced by *Desulfomonile* and Desulfobacteraceae group Sva0081. *Desulfomonile* was only present in a narrow horizon, while Sva0081 persisted throughout the depth of the mat. *Desulfovibro* and *Syntrophobacter* are known to oxidize organic substrates incompletely to acetate, while *Desulfomonile* and most members of the family Desulfobacteraceae can perform complete oxidation of organic substrates, including acetate, to CO_2_ (*43*). Thus, the community stratigraphy reflected a progressive, systematic shift in carbon fixation and remineralization potential along a depth profile through the mat. Microbial diversity was lowest at the surface but increased with depth to a maximum ~4 cm—just below the transition between groups of sulfate reducers (Fig. 2, G and H). This could reflect the availability of small organic substrates used by a greater diversity of heterotrophs.

Fluorescence microscopy on a mat microtome section embedded in Steedsman’s wax to preserve spatial relationships illustrated its characteristic palisade texture defined by upward radiating sheathes of large filamentous Cyanobacteria with a heterogeneous distribution of other bacteria (Fig. 2I). Despite this microscopic heterogeneity, our depth profile resolved clear patterns in millimeter- to centimeter-scale community structure, and replicate samples showed similar trends (Fig. 2C). Therefore, while microenvironments undoubtedly control microbial ecology from the perspective of individual cells, many aspects of overall ecosystem function can be appreciated from a much coarser picture.

This characterization of microbial community composition with depth provided us a framework for understanding the Little Ambergris Cay mat ecosystem, with niches partitioned along gradients of light and chemistry. Although we do not have functional insight into much of the extraordinary diversity in these communities, the taxa highlighted here served as windows into processes of carbon, oxygen, and sulfur cycling, providing us with indicators to gauge ecosystem function and recovery in the aftermath of Hurricane Irma.

### Community variance across space and time

**Fig. 3.**
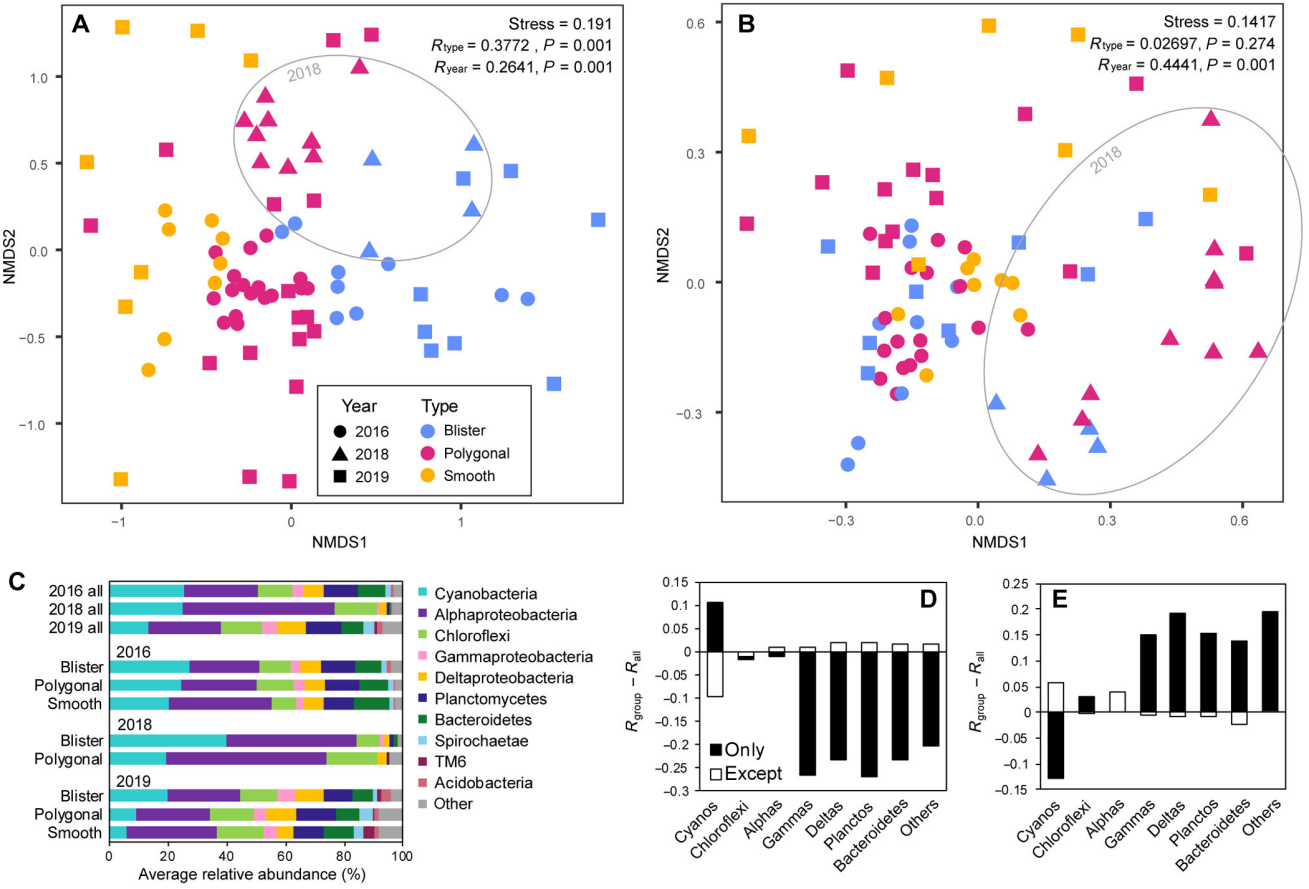
Differences in bulk mat communities across space and time. (**A**) NMDS plot visualizing variance, showing a clear trend between mat types along with a perturbation following the hurricane that largely recovered by 2019. Additional metadata variables can be found in figs. S2 and S7. (**B**) NMDS plot of phylum level rather than OTU level data. At the phylum level, the trend between mat types is lost, but the perturbation in 2018 remains clear. (**C**) Average phylum level community composition of all bulk mat samples from each year and of each mat type within each year. The individual samples included in these averages can be found in fig. S3. (**D** and **E**) The contribution of each major group of organisms to the community variance seen between mat types (D) and year (E), quantified as the difference in ANOSIM statistic *R* between the full dataset and the dataset filtered to include only a specific group of organisms (shaded bars) and the dataset filtered to exclude that specific group (open bars). NMDS plots accompanying these calculations can be found in fig. S4. The aspects of the community that varied most strongly between mat types and those most strongly perturbed by the hurricane are notably distinct—almost inverse.

To understand the biological differences between the distinct mat types on Little Ambergris Cay, we surveyed bulk mat communities across different mat morphologies and locations. Two areas featured in this survey, LB and F, included transects across all three mat types (fig. S1). Broadly, the mat communities across the island were all similar, but NMDS and analysis of similarities (ANOSIM) resolved patterns within the observed variance (Fig. 3). While there was no correlation between community composition and location on the island (*R* = 0.0558, *P* = 0.094) (fig. S2B), there was a clear trend between the different mat types (*R* = 0.3772, *P* = 0.001) corresponding to their relative elevations, with polygonal mats sitting between smooth and blister mats (Fig. 3A). We repeated this survey 10 months (2018) and 21 months (2019) following Hurricane Irma and saw a shift in the mat communities between the prehurricane (2016) and 2018 datasets that largely recovered by 2019. The variance between mat types and the variance between years are expressed along different vectors in the NMDS plot, suggesting that different aspects of the community contribute to the between-mat–type differences and the between-year differences.

At the phylum level, the microbial communities of the different mat types are indistinguishable, demonstrating that community differences between mat types occurred only on finer taxonomic scales (Fig. 3, B and C). In contrast, the perturbation in 2018 is quite clear—the Cyanobacteria, Alphaproteobacteria, and Chloroflexi remained abundant, while other major phylum level groups were considerably diminished.

To explore how the variance in this dataset was expressed within major groups, we conducted NMDS and ANOSIM analyses on each phylum level group individually, with a corresponding dataset of the remainder of the community excluding each group (fig. S4). By comparing the ANOSIM statistic *R* values calculated with these subsets to those calculated with the full communities, we obtained a measure of which groups contributed most strongly to the differences between mat types and years. The Cyanobacteria were the only group to contribute substantially higher than average to the differences between mat types, with the Cyanobacteria alone exhibiting clear variance between mat types (*R* = 0.4824, *P* = 0.001) and the remaining dataset excluding Cyanobacteria exhibiting considerably less variance between mat types (*R* = 0.2815, *P* = 0.001) (Fig. 3D). The Chloroflexi and Alphaproteobacteria exhibited variance between mat types comparable to the full communities, and all the other groups exhibited considerably less variance between mat types. In contrast, the groups that exhibited differences between years—reflecting the hurricane impact—were almost the inverse of those that exhibited differences between mat types. Cyanobacteria exhibited the least variance between years (*R* = 0.1514, *P* = 0.002), the Chloroflexi and Alphaproteobacteria were roughly average again, and the other groups exhibited much higher variance between years, with the highest from the Deltaproteobacteria (*R* = 0.4709, *P* = 0.001) (Fig. 3E).

**Fig. 4.**
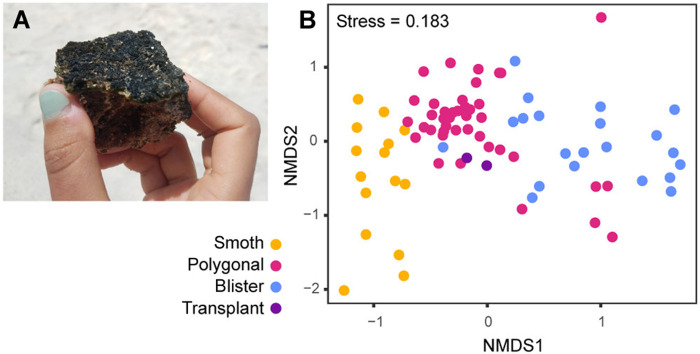
Transplant experiment. (**A**) Photograph of a transplanted piece of polygonal mat, turned dark and hard—reminiscent of blister mat—1 year after transplantation. (**B**) NMDS plot of only cyanobacterial OTUs, illustrating their clear pattern of variance between mat types. Transplant samples are more similar to polygonal mats than blister mats, indicating that mat texture reflects environmental factors more than community composition and demonstrating marked tolerance for environmental change from these taxa.

Since the distribution of mat types is controlled by exposure time above water (*32*), we hypothesized that the aspects of the communities that differed most strongly between the mat types (the Cyanobacteria) reflected those most sensitive to sea level. To investigate the response of these taxa to a change in relative water level, we transplanted a slice of polygonal mat such that its surface sat several centimeters higher—into the elevation range that tends to host blister mats. One year later, the transplanted mat had developed a hard, darkly pigmented surface, resembling a blister mat (Fig. 4A). However, the cyanobacterial community members remained comparable to those found in polygonal mats (Fig. 4B). This experiment suggested that although the cyanobacterial populations between blister and polygonal mats are generally distinct, the physical expression of mat morphology primarily reflects environmental context, not simply microbial community differences. Despite demonstrating clear environmental preference in their naturally occurring distributions, these populations persisted outside of their preferred range over the course of this 1-year experiment.

### Rapid posthurricane new mat growth

**Fig. 5.**
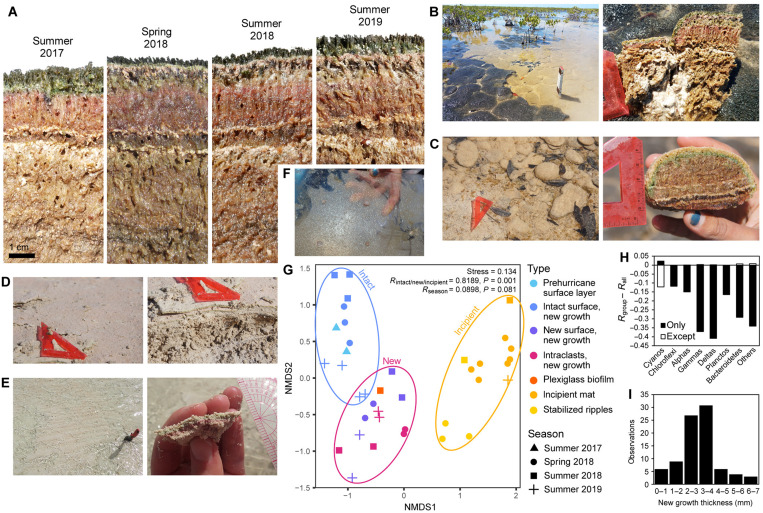
Posthurricane growth. (**A**) Photographs of the CC mat depth profile through time, beginning 1 month before the hurricane in August 2017. This area was well protected by mangroves and therefore affected minimally. A thin layer of sediment deposited by the storm is visible in all subsequent photographs, with increasing new mat growth above it. Sediment lags several centimeters below the mat surface could represent previous storm events, possibly Hurricanes Ike in 2008 and Frances in 2004. (**B**) Scoured polygons adjacent to intact polygons in the CC area. New growth is visible on both intact and scoured surfaces. (**C**) Intraclasts of ripped up mat that were rounded during transport and redeposited upside down at MCW. (**D**) Incipient mat on top of hurricane sediment at FM. (**E**) Microbially stabilized ripples at BB. (**F**) Plexiglass biofilm experiment at CC. (**G**) NMDS plot of posthurricane growth samples. (**H**) ANOSIM analysis showing the contribution of different groups to the variance between new growth types. (**I**) Histogram showing thicknesses of new growth from 86 measurements of upside down mat intraclasts observed in March 2018, 6 months after the hurricane.

Both scour and sediment deposition during Hurricane Irma decimated large areas of mat. However, new mat growth developed rapidly over the surfaces exposed or deposited by the hurricane (Fig. 5). We categorized this new growth into three types based on the degree of perturbation—new growth on intact mat surfaces with minimal hurricane sediment (Fig. 5, A and B); new growth on new mat surfaces such as where the old mat surface had been scoured out (Fig. 5B) or on intraclasts of ripped up mat that had been redeposited upside down (Fig. 5, C and I); and incipient growth on/in hurricane sediment deposits (Fig. 5D), sometimes stabilizing sedimentary bedforms such as ripples (Fig. 5E). We monitored the development of these posthurricane growth types 6 months (March 2018), 10 months (July 2018), and 21 months (June 2019) after the hurricane.

Community analyses showed that the variance among the posthurricane growth samples was neatly grouped by the intact/new/incipient types (*R* = 0.8189, *P* = 0.001) (Fig. 5G). However, the variance did not show a trend through time (*R* = 0.0898, *P* = 0.081), which would reflect ecological succession. This suggested that if there was any succession in the establishment of this new growth, it occurred on a time scale not resolved by our field sampling campaigns—within 6 months after the hurricane. Furthermore, a short-term growth experiment [a biofilm developed on a sheet of plexiglass deployed in the field for 1 week in 2018 (Fig. 5F)] yielded a microbial community very similar to the other new growth samples, demonstrating that this complex community was able to colonize a fresh surface extremely rapidly. However, this experiment did not repeat in 2019 (no visible biofilm developed), suggesting that growth conditions during the aftermath of the hurricane were different from steady state.

Similar to what we observed among the original mat types, we found that the Cyanobacteria exhibited the strongest contribution to the community variance between new growth types (*R* = 0.8411, *P* = 0.001) (Fig. 5H). The rapid development of these communities in colonizing new surfaces suggested that they are very robust and dynamic in their ability to respond to environmental disruption. Together with their persistence in our transplant experiment, this suggested that the community is highly resistant to perturbation. Perturbation tolerance is thought to correlate with high biodiversity (*45*); we recovered over 6000 cyanobacterial operational taxonomic units (OTUs), with abundant representatives from four of the five cyanobacterial subsections documented by both DNA sequence and morphology (fig. S6).

### Slower recovery of the microbial sulfur cycle

**Fig. 6.**
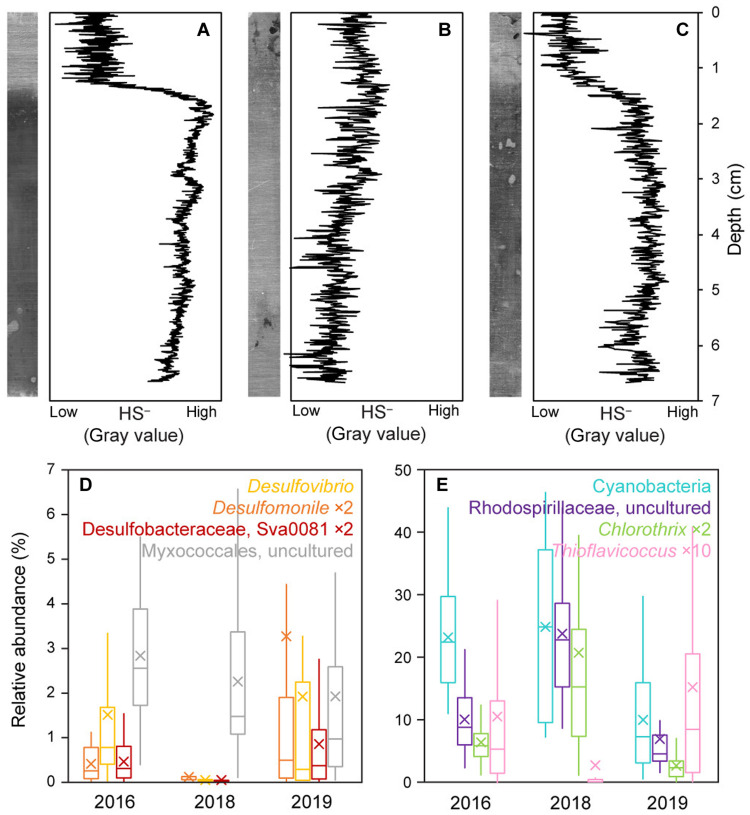
The sulfur cycle recovered on a slower time scale than new mat growth. (**A** to **C**) Sulfide profiles captured on silver strips at CC depth profile site in (A) 2017, (B) 2018, and (C) 2019, shown as both raw scanned images (left) and quantified by gray value (right). In 2017 and 2019, there was a clear sulfidic zone in the subsurface mat. In 2018, the year following Hurricane Irma, the mats did not appear meaningfully sulfidic at any depth. (**D** and **E**) Box and whisker plots showing the relative abundances of major Deltaproteobacteria (D) and phototrophs (E) in bulk mat samples from each year. Boxes denote first and third quartiles, horizontal lines indicate medians, x’s indicate averages, and whiskers indicate minimum and maximum data points. Taxa implicated in sulfate reduction or sulfide oxidation were substantially diminished in 2018, consistent with an impacted sulfur cycle.

Although robust new mat growth was evident as early as 6 months after the hurricane, it was also clear from field observations that even where the mats remained intact, the hurricane had markedly affected their biogeochemistry. Most notably, there was no perceptible odor of sulfide (which can be detected by smell in amounts as low as 0.008 parts per million (*46*)), which had been a ubiquitous characteristic of the mats before the hurricane. Silver strips inserted into the mats to capture sulfide profiles (*22*) confirmed the absence of sulfidic porewater (Fig. 6B). A sulfide profile similar to the prehurricane baseline only returned by our 2019 field campaign (Fig. 6C). These observations suggested that the hurricane impact temporarily disrupted the sulfur cycle within the mats.

Using specific taxonomic groups as indicators of ecosystem processes provided another line of evidence for a disrupted sulfur cycle in 2018. Within the Deltaproteobacteria—a group that exhibited particularly high variance between years (Fig. 3E)—all three major genera of sulfate-reducing bacteria were diminished in 2018, while the aerobic Myxococcales remained abundant throughout (Fig. 6D). Similarly, among the major phototrophs, the Cyanobacteria and photoheterotrophs (uncultured member of the Rhodospirillales and *Chlorothrix* of the Chloroflexales) remained abundant throughout, while the sulfide-oxidizing Chromatiales (*Thioflavicoccus*) were diminished in 2018 (Fig. 6E). All of these taxa recovered by 2019, along with the reestablishment of a measurable sulfide concentration profile. Together, the lack of sulfide and absence of both sulfate-reducing and sulfide-oxidizing bacteria in 2018 demonstrate, by both function and community composition, that sulfur cycling within the mats stalled during the year following Hurricane Irma.

## DISCUSSION

In ecological theory, perturbations are classified as pulses—discrete, relatively instantaneous alterations—or presses—sustained, gradual alterations (*47*, *48*). Global climate change is, by definition, a press; however, it also increases the frequency and severity of pulses, including but not limited to extreme storm events such as Hurricane Irma (*41*, *49*). These different types of perturbation tend to carry different patterns of microbial community response, and the impacts of multiple perturbations may interact with each other in complex ways (*50*). Therefore, understanding the ecological implications of climate change requires understanding how each type of perturbation affects communities, the extent to which communities can recover from them, and how they might influence each other. The dataset presented here has implications for both pulse (Hurricane Irma) and press (sea level rise) perturbations on a coastal microbial mat ecosystem.

Our depth profile characterization described an ecosystem governed by carbon cycling through primary producers and decomposers (and secondary and tertiary decomposers) and sulfur cycling through both producers and consumers of sulfide. In many ways, this nutrient cycling is the microbial equivalent of the trophic levels that comprise classical macrofaunal ecosystems; rather than predator/prey relationships, species interactions are based primarily on the production and consumption of chemical substrates. The hurricane severely disrupted the chemical gradients that enabled many of those interactions. The rapid development of new growth in the wake of the hurricane reflected the populations not dependent on those gradients or the buildup of certain substrates—phototrophs, aerobic heterotrophs, and metabolically flexible mixotrophs—but lacked many of the niche spaces available in the climax community, exemplified by the absence of sulfur cycling taxa and a sulfidic chemocline. By analogy to classical ecology, the populations dependent on an intricate food web (or higher trophic levels) lagged behind the initial community. The subsequent return of sulfate-reducing and sulfide-oxidizing bacteria along with a sulfidic chemocline illustrated the recovery of biogeochemical cycling characteristic of a mature mat ecosystem.

The sulfur cycle has important connections to the carbon storage potential of mangrove and mat ecosystems. Reactions between dissolved sulfides and organic matter have been implicated in decreasing organic matter lability and thereby increasing its preservation potential. This phenomenon is known to occur in the Little Ambergris mats (*51*) and has been suggested to account for as much as half of the organic matter preservation associated with mangrove forests (*52*)—ecosystems noted for their disproportionately important contributions to global carbon storage and therefore targeted by restoration and conservation efforts aimed at ameliorating anthropogenic carbon emissions (*53*). In the absence of sulfides generated by microbial sulfate reduction, these sulfurization reactions are unlikely to occur. Therefore, although the mat sulfur cycle ultimately recovered from the hurricane impact, the interruption that we observed likely carries consequences in the form of lost carbon storage potential. This means that the expected increase in extreme storm events due to climate change may have adverse implications for the carbon sequestration capacities of mangrove and mat ecosystems.

Since the sulfur cycle disruption was seen even in mats that remained fully intact, this aspect of the hurricane impact was likely due to the extreme degree of fluid inundation flushing away soluble substrates and overwhelming anaerobic communities with oxic waters rather than physical disruption of mat integrity or burial. That being said, the sediment underlying the Little Ambergris mats comprises primarily ooid sand grains, which approximate close-packed spheres and therefore accommodate substantial pore space that promotes fluid permeability. This means that considerable flushing likely accompanies normal tidal cycles, introducing oxic seawater and moving soluble nutrients (*34*), and the gradients powering mat biogeochemical function are robust enough to weather that degree of flushing. Therefore, the flushing induced by Hurricane Irma must have exceeded some critical threshold in their O_2_-buffering capacity. Irma was the strongest hurricane ever to hit Little Ambergris Cay in recorded history, although the island experiences hurricane force winds on average once every 5.5 years (*54*), and tropical storms more frequently than that. A better understanding of where this threshold sits on the continuum from normal daily tidal flushing to Hurricane Irma is required to appreciate the severity of these implications for changes going forward.

In contrast to the posthurricane rapid colonization of fresh surfaces and reestablishment of gradients in surviving mats, adaptation to changing sea level requires mats in a given location to shift from one type to another as relative water level shifts around them. For the community differences among mat types to persist, taxa that are specific to a given mat type—and therefore likely well adapted to the narrow habitat ranges that distinguish them—will have to migrate into areas that previously hosted a different mat type. However, our transplant experiment demonstrated impressive persistence of a polygonal mat community in the environmental context of a blister mat. This suggests that although mat morphologies will shift with changing sea level, established mat communities that can tolerate the change may exhibit priority effects, inhibiting the immigration of exogenous taxa that would otherwise be better adapted to that specific environment (*48*, *55*). Nonetheless, we observed posthurricane new growth analogous to the full range of mat types—with analogous community differences—after the hurricane had scoured out or buried much of the mat area. This new growth occurred at a much higher rate than steady-state mat growth, suggesting that the hurricane perturbation enabled the new growth, perhaps by resetting whatever factors limit growth, creating fresh surfaces for colonization, or aiding in dispersal. It is possible that by disrupting the invasion-resistant established mat communities and promoting the redistribution of taxa, these perturbations could facilitate the development of mat communities most optimized to a given habitat range. Therefore, the occurrence of pulse disturbances such as a hurricane may enable adjustment to the press disturbance of sea level change for this ecosystem, exemplifying the complex effects of multiple simultaneous forcing factors.

Together, this study demonstrates the substantial resilience of Little Ambergris Cay microbial mats in the face of both pulse and press disturbances induced by climate change. The mat communities and putative biogeochemical functions largely recovered from Hurricane Irma—a markedly destructive perturbation—within 2 years. In contrast, catastrophic hurricanes threaten extinction for island macrofauna with limited reproduction rates and dispersal abilities (*56*). While this robustness in the face of environmental perturbation is consistent with the geological record of microbial mat ecosystems persisting through past intervals of climate change, this study resolved a granularity that can only be observed in the modern and rates of both perturbation and recovery that likely exceed most historical examples.

## MATERIALS AND METHODS

### Fieldwork

We conducted five field campaigns, in July 2016, August 2017, March 2018, July 2018, and June 2019, with an additional campaign limited to drone imaging immediately following the hurricane in September 2017. We conducted an initial bulk mat survey to understand the diversity of microbial mats across the island in July 2016 and focused on the depth profile at a single mat site in August 2017. Following Hurricane Irma, we surveyed new growth types in the March 2018, July 2018, and June 2019 field seasons and repeated the 2016 bulk mat survey in July 2018 and June 2019.

### Drone photography

Aerial imaging was done using a DJI Phantom 4 Pro uncrewed aerial vehicle equipped with a built-in 12 megapixel complementary metal-oxide semiconductor camera. Full island orthomosaics were generated using Agisoft Photoscan software from overlapping nadir images, as described by Stein *et al*. (*32*).

### Mat sampling

Mat samples were collected using ethanol sterilized razor blades or spatulas into BashingBead lysis tubes containing a DNA preservation buffer (Zymo). Because of the manufacturer changes over the course of this study, two different preservation buffers were used. The 2016 samples were preserved in Xpedition lysis/stabilization solution; 2017 and 2018 samples were preserved in DNA/RNA Shield. In 2019, we collected a set of samples with replicates in each buffer to constrain artifacts introduced by changing the buffer. See fig. S7 for a more detailed discussion of this buffer discrepancy.

### DNA extraction, amplification, and sequencing

DNA was extracted at Caltech using the Zymo Quick-DNA Fecal/Soil Microbe MiniPrep kit. A segment of the V4 to V5 hypervariable region of the 16*S* rRNA gene was amplified by polymerase chain reaction (PCR) using the 515f and 926r primer pairs (*57*). PCR reactions were set up in 15 μl volumes with Q5 Hot Start High-Fidelity 2× Master Mix (New England Biolabs), with annealing at 54°C and 30 cycles. Amplified products were barcoded with Illumina NexteraXT index 2 primers, and barcoded samples were submitted to Laragen for 2 × 250–bp paired-end sequencing on Illumina’s MiSeq platform.

### Amplicon sequence data processing

Sequence data were processed using QIIME version 1.8.0 (*58*). Raw sequence pairs were joined and quality trimmed. Sequences were then clustered into OTUs with 99% similarity using the UCLUST open reference clustering protocol, and the most abundant sequence was chosen as representative for each OTU. Taxonomic identification for each representative sequence was assigned using the Silva-119 database (*59*), and community composition tables at the OTU, genus, order, and phylum level, with both absolute and relative abundance were generated. Unless otherwise specified, analyses were conducted on the OTU level. OTUs that were taxonomically unassigned, singletons, assigned to the Eukaryota, or likely contaminants indicated by abundance in a negative control were removed. Samples that returned fewer than 1000 sequence reads were not included in analyses. NMDS and ANOSIM analyses were done by calculating a Bray dissimilarity matrix using the vegan ecology package in R (*60*). Diversity indices were calculated on datasets rarefied to 3000 reads.

### Sulfide profiles

Sulfide profiles were captured on clean, polished silver strips inserted into the mats similar to the method described by Fike *et al*. (*22*). The strips were left to react for 1 hour and then gently rinsed off and wrapped in Kimwipes to avoid disrupting the silver sulfide precipitated on the surface. Upon return to Caltech, the strips were imaged with a flatbed scanner, and the profile of captured silver sulfide was quantified by gray value in ImageJ along a straight vertical path chosen to minimize encounters with bubbles or other anomalies. We note that this method does not capture sulfide that is lost by reoxidation under oxic conditions; therefore, the profiles captured here are interpreted as a demonstration of the sulfidic chemocline rather than as an absolute determination of sulfide concentration with depth.

### Microscopy

Light microscopy was conducted during fieldwork on wet mounts using an Amscope B120 LED microscope equipped with an Amscope MD500 eyepiece camera. For fluorescence microscopy, mat samples were fixed in 4% paraformaldehyde in phosphate-buffered saline (PBS) for 1 hour, washed in PBS, dehydrated with 15-min incubations in a series of increasing ethanol:PBS solutions (50:50, 70:30, and 90:10), and stored in 100% ethanol. Upon return to the laboratory at Caltech, the fixed mats were embedded in Steedman’s wax and sliced with a microtome into 5- to 10-μm sections, which were deposited onto Suprafrost Plus microscope slides (Thermo Fisher Scientific). The wax was dissolved with three 5-min incubations in 100% ethanol. The remaining biomass was fluorescently labeled with the universal bacterial probe combination EUB338mix (EUB338, -II, and -III) with the fluorescein isothiocyanate (FITC) fluorophore (Integrated DNA Technologies). Fluorescence in situ hybridization (FISH) was carried out at 35% formamide concentration as described by McGlynn *et al*. (*61*). Biomass was also counterstained with 4′,6-diamidino-2-phenylindole (DAPI; 4.5 ng/μl) in Citifluor AF1 mounting medium. Tiled fluorescent images were produced on a Zeiss Elyra PS.1 using a Plan-APOCHROMAT 100×/1.46 Oil DIC M27 objective. DAPI, FITC, and cyanobacterial autofluorescence were illuminated with 405-, 488-, and 561-nm laser lines and viewed through BP420-480 + LP750, BP495-550 + LP750, and BP570-620 + LP750 filter sets, respectively.

### Transplant experiment

During summer 2018 fieldwork, a several-centimeter-thick piece of CC mat (excised to take the evolving depth profile photograph shown in Fig. 5) was left sitting on top of the polygonal mat surface such that the surface of the excised mat was several centimeter higher than before. When we returned in 2019, this transplanted mat remained undisturbed. We sampled it to explore any changes in the microbial community.

### Plexiglass biofilm experiment

During summer 2018 fieldwork, a sheet of plexiglass was deployed at the CC site, secured by zip tie to mangroves and tent stakes. After 1 week, a biofilm that had developed on the surface was sampled with an ethanol-sterilized paintbrush. Attempts to repeat this experiment in 2019 failed—no visible biofilm developed.

## Notes

View/request a protocol for this paper from *Bio-protocol*.
