# Supplementary material for: Early impacts of climate change on a coastal marine microbial mat ecosystem
Source: Sci Adv. 2022 May 27;8(21):eabm7826. doi: 10.1126/sciadv.abm7826 (PMC9140962; doi:10.1126/sciadv.abm7826)
Supplement: Supplementary file 1 — Figs. S1 to S7 [file sciadv.abm7826_sm.pdf]

Science Advances
AAAS

Supplementary Materials for

# Early impacts of climate change on a coastal marine microbial mat ecosystem

Usha F. Lingappa *et al.*

Corresponding author: Usha F. Lingappa, usha@caltech.edu

*Sci. Adv.* **8**, eabm7826 (2022)
DOI: 10.1126/sciadv.abm7826

**This PDF file includes:**

Figs. S1 to S7

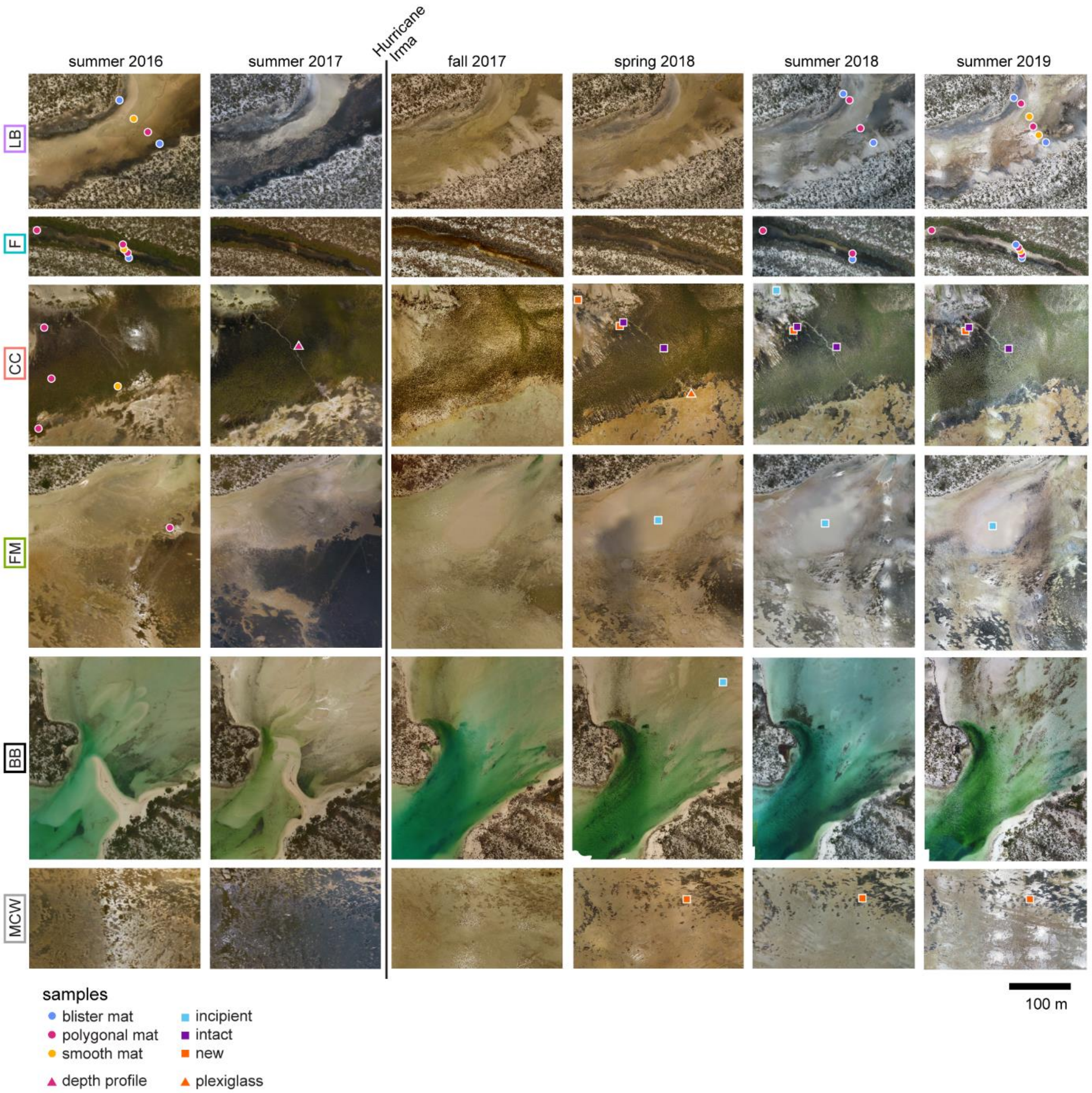


**Figure S1.** Drone images of mat study areas showing aerially visible changes over time. Specific sample locations from each field season are marked; some markers represent multiple samples of the same type taken in close proximity. The LB and F areas featured all three endmember mat types along transects of varying water depth across channels. The CC area contained thick, lush polygonal mats largely protected by mangrove thickets. The FM area hosted extensive mats prior to Hurricane Irma, most of which were scoured out or buried under a large lobe of sediment deposited during the storm, the surface of which subsequently developed incipient growth. The BB area also experienced massive changes in sedimentation from the hurricane, and transiently featured microbially-stabilized ripples. MCW was one of many areas that accumulated deposits of mat intraclasts after the hurricane.

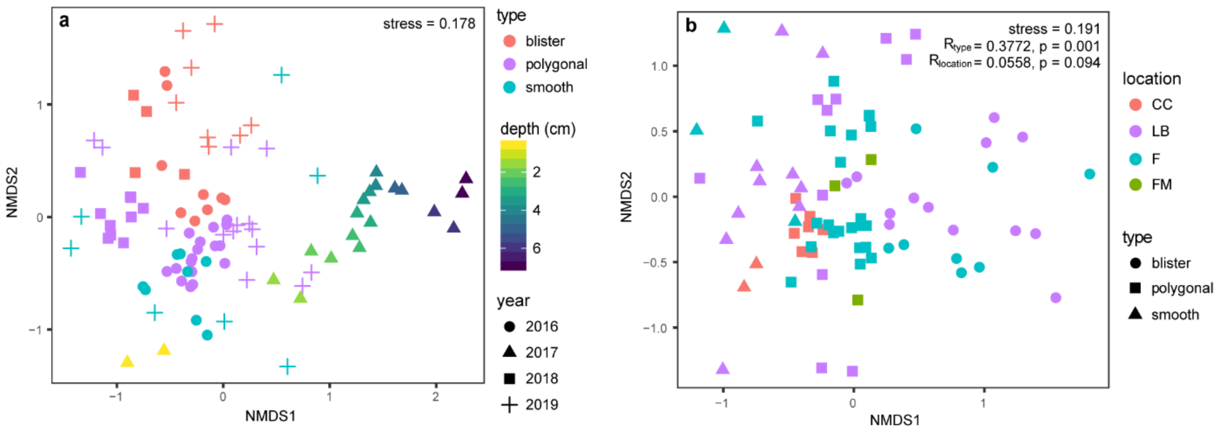


**Figure S2.** Additional NMDS plots of variance in bulk mat dataset. ***A***, Bulk samples plotted with the depth profile dataset. The variance between bulk mats plotted along a different vector than the variance with depth, indicating that the variance between bulk mats was not merely a reflection of how much of the subsurface community was sampled. ***B***, NMDS plot from Figure 3*A* colored by sample location, which did not exhibit a meaningful trend.

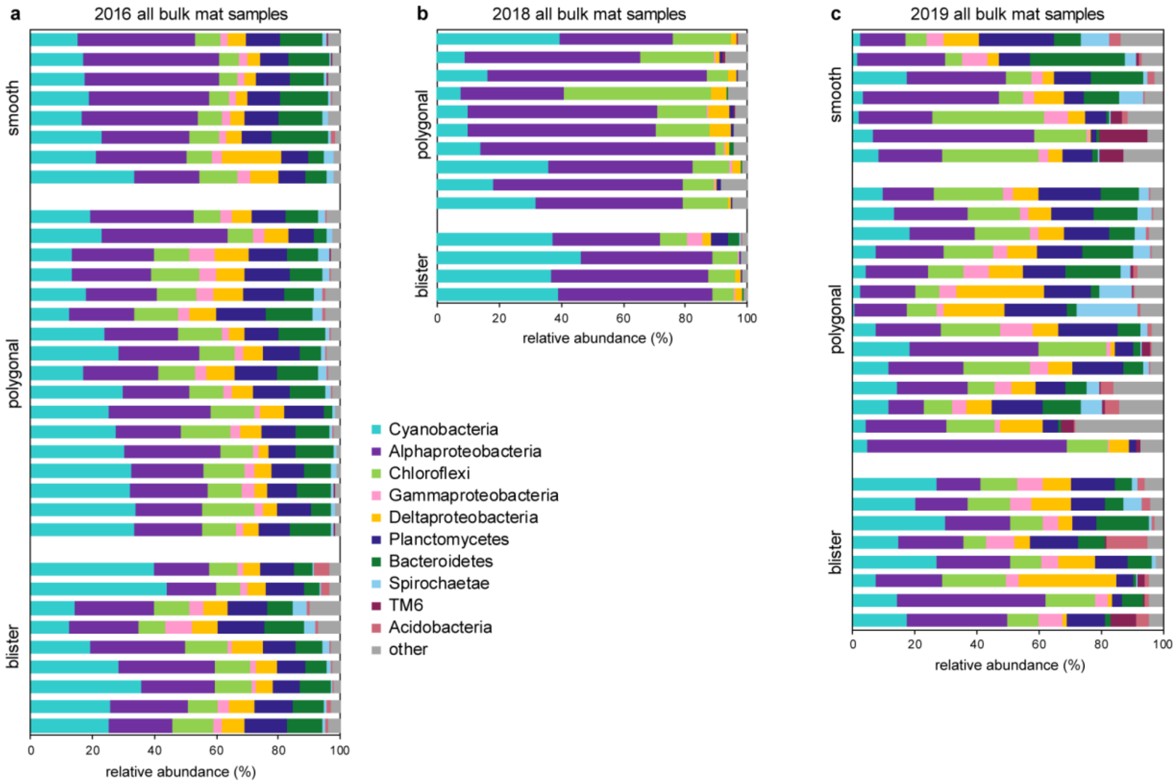


**Figure S3.** Phylum level community composition of the individual bulk mat samples from ***A***, 2016, ***B***, 2018, and ***C***, 2019 that contributed to the averages shown in Figure 3*C*.

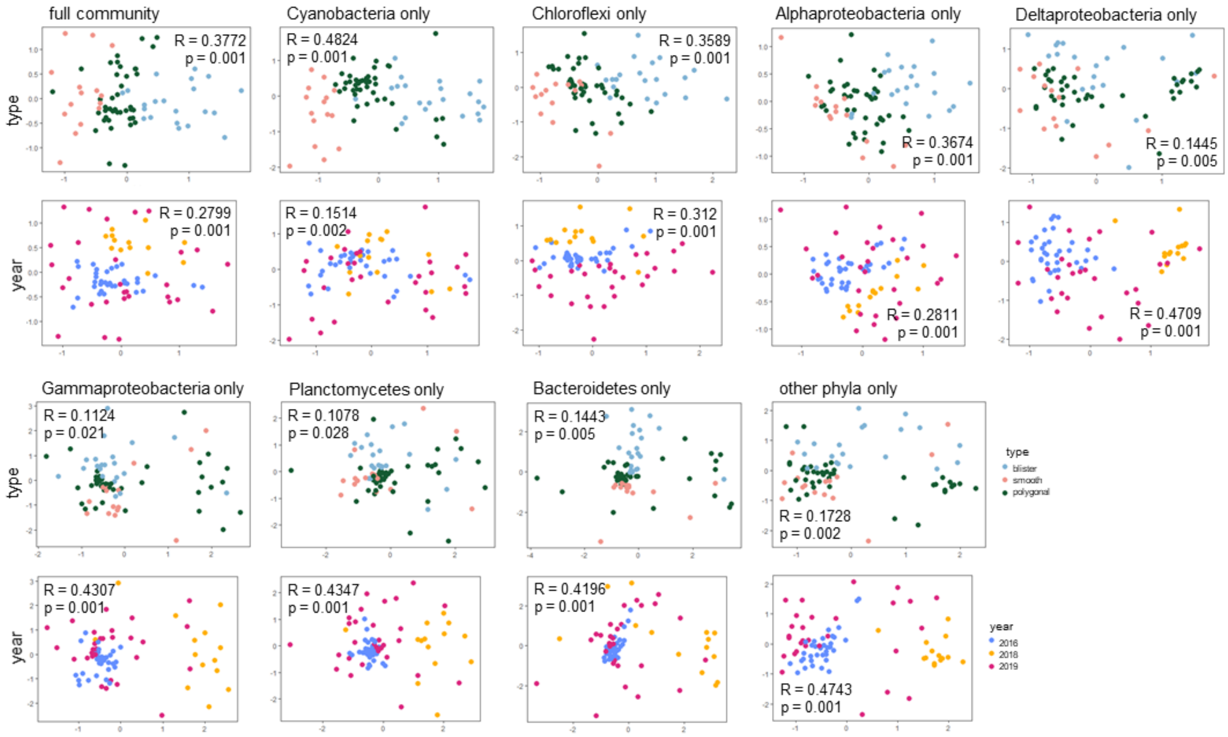


**Figure S4.** NMDS plots visualizing variance within major groups of organisms from the subsetted datasets used to calculate the ANOSIM values in Figure 3*D-E*. The plot for each group is shown twice with the points colored by mat type or year, to highlight how different groups express patterns in dissimilarity for each variable. ANOSIM statistic *R* values are displayed for the variable in question.

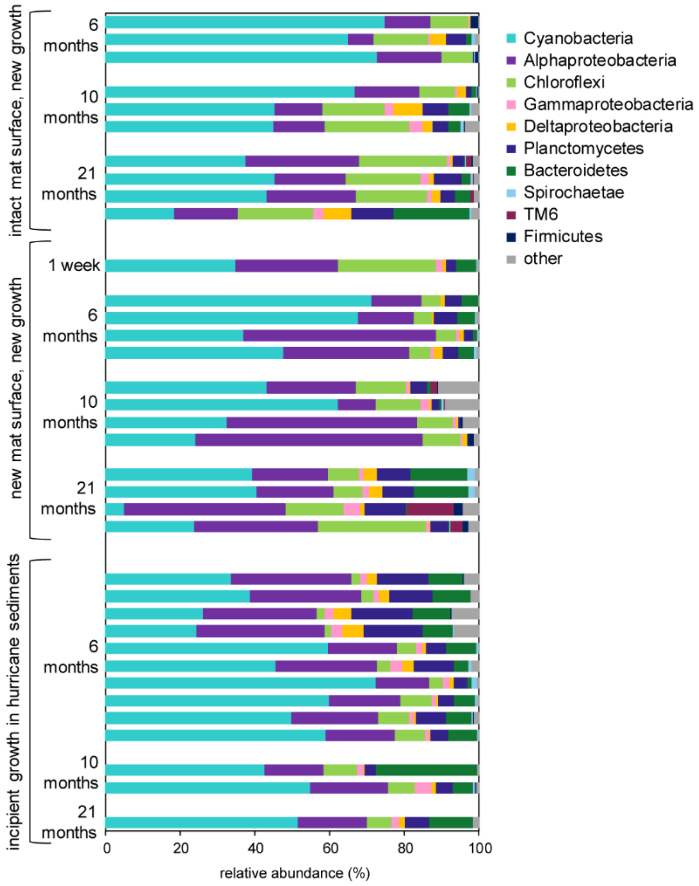


**Figure S5.** Phylum level community composition of post-hurricane growth samples.

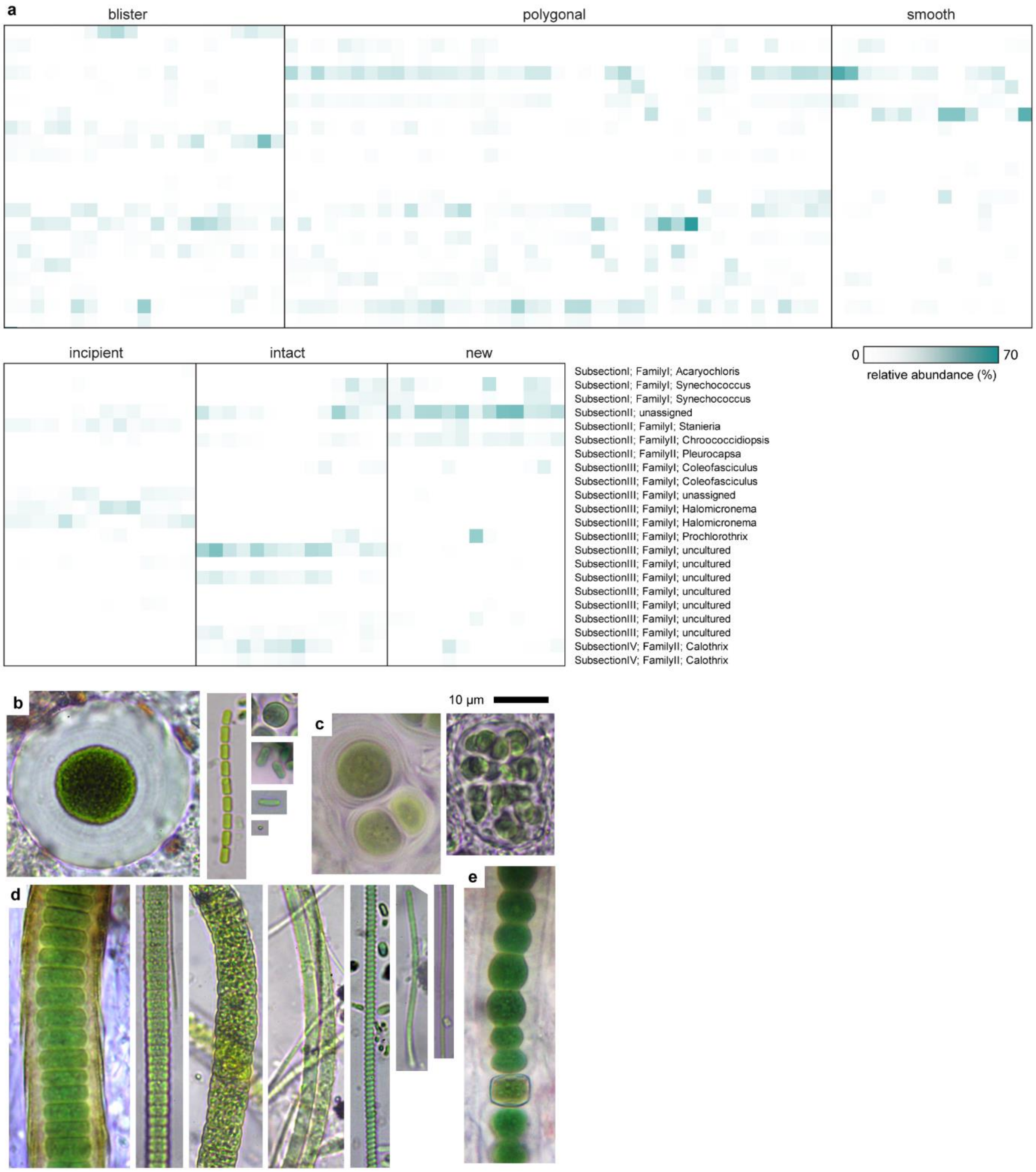


**Figure S6.** Diversity of Cyanobacteria in the Little Ambergris Cay mats. ***A***, Heat map showing relative abundance of the top cyanobacterial taxa (OTUs with average > 0.5% of cyanobacterial reads in a sample), together accounting for 36% of all cyanobacterial reads. ***B-E***, Photomicrographs illustrating the morphological diversity of Cyanobacteria in the Little Ambergris Cay mats, including unicellular (***B***), colonial/baeocystous (***C***), filamentous (***D***), and heterocystous (***E***) representatives.

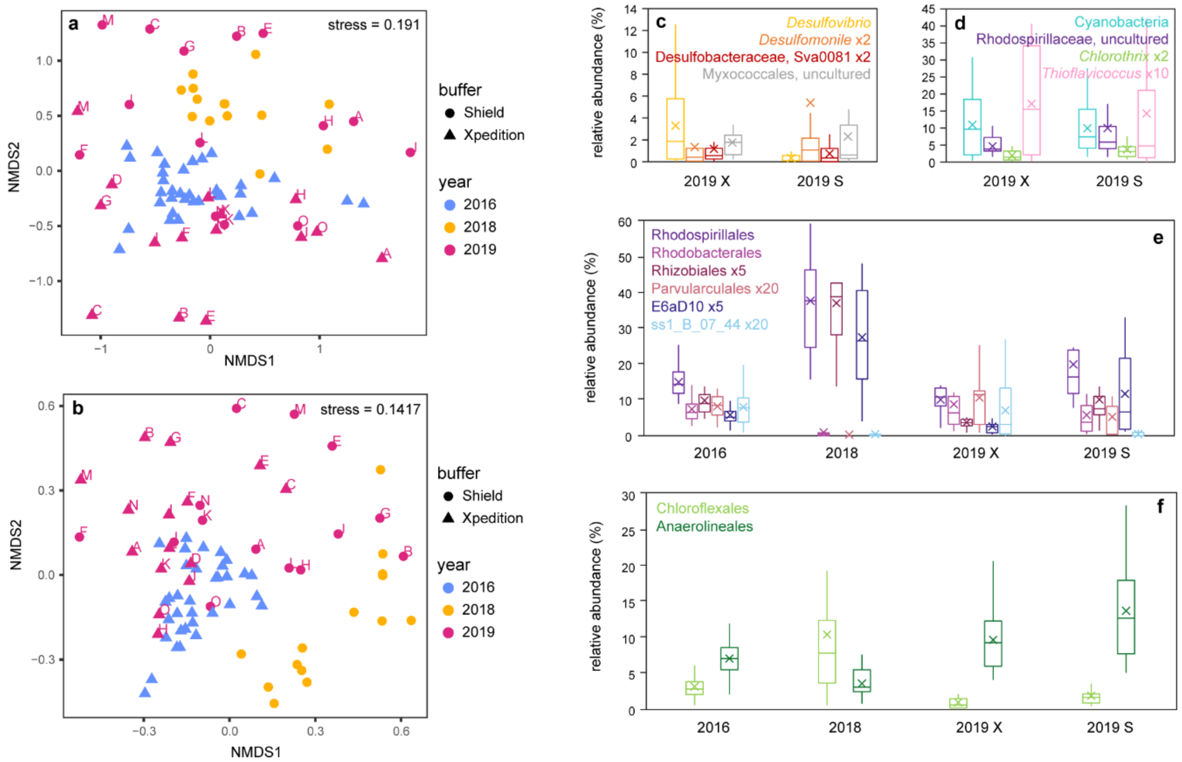


**Figure S7.** Deconvolving the buffer artifact from the hurricane impact. ***A-B***, NMDS plots from Figure 3 at the OTU (***A***) and phylum (***B***) levels, showing which DNA preservation buffer was used. Letters indicate 2019 replicates. The buffer discrepancy complicated interpretation of the variance between years at the OTU level, but phylum level data showed a clear pattern of hurricane impact and recovery regardless of buffer. ***C-D***, Box and whisker plots of the taxa shown in Figure 6, comparing samples collected in 2019 with the Xpedition (X) buffer used in 2016 and the Shield (S) buffer used in 2018. There was a possible buffer artifact in the Desulfovibrionales, but the remainder of these taxa seemed unaffected by buffer, strengthening our interpretation that the perturbation illustrated with these taxa represented real ecological change. ***E-F***, We also examined major orders within the Alphaproteobacteria and Chloroflexi, two of the most dominant groups across all samples. ***E***, Within the Alphaproteobacteria, some orders were clearly diminished (Rhodobacterales, Parvularculales) while others were enriched (Rhodospirillales, Rhizobiales) in 2018; these taxa did not seem affected by buffer, suggesting that their perturbation also reflected the hurricane impact. However, they are sufficiently functionally diverse that it is inadvisable to draw specific biogeochemical conclusions from their presence or absence. Other groups (E6aD10 and ss1_B_07_44) did exhibit buffer effects. ***F***, Within the Chloroflexi, both the phototrophic Chloroflexales and anaerobic, fermentative Anaerolineae were abundant throughout our timeseries, but their relative dominance was switched in 2018. These taxa did not exhibit a buffer effect.
